# Factor XII–driven coagulation traps bacterial infections

**DOI:** 10.1084/jem.20250049

**Published:** 2025-04-22

**Authors:** Katrin F. Nickel, Anne Jämsä, Sandra Konrath, Praveen Papareddy, Lynn M. Butler, Evi X. Stavrou, Maike Frye, Mathias Gelderblom, Bernhard Nieswandt, Sven Hammerschmidt, Heiko Herwald, Thomas Renné

**Affiliations:** 1 Institute of Clinical Chemistry and Laboratory Medicine, University Medical Center Hamburg-Eppendorf, Hamburg, Germany; 2 Clinical Chemistry, Department of Molecular Medicine and Surgery, and Center of Molecular Medicine, Karolinska Institutet and University Hospital, Stockholm, Sweden; 3 Clinical Chemistry, Medical Diagnostics, Karolinska University Hospital, Stockholm, Sweden; 4 Department of Laboratory Medicine, Biomedical Center (BMC), Lund University, Lund, Sweden; 5 Department of Clinical Medicine, The Arctic University of Norway, Tromsø, Norway; 6 Science for Life Laboratory, Department of Protein Science, Royal Institute of Technology (KTH), Stockholm, Sweden; 7 Medicine Service, Section of Hematology-Oncology, Louis Stokes Veterans Administration Medical Center, Cleveland, OH, USA; 8 Department of Medicine, Hematology and Oncology Division, Case Western Reserve University School of Medicine, Cleveland, OH, USA; 9 German Centre of Cardiovascular Research (DZHK), Partner Site Hamburg/Luebeck/Kiel, Hamburg, Germany; 10 Department of Neurology, University Medical Center Hamburg-Eppendorf, Hamburg, Germany; 11 Institute of Experimental Biomedicine, Chair of Experimental Biomedicine I, University Hospital Würzburg, Würzburg, Germany; 12 Rudolf Virchow Center for Integrative and Translational Bioimaging, Julius-Maximilians-Universität Würzburg, Würzburg, Germany; 13 Department of Molecular Genetics and Infection Biology, Interfaculty Institute of Genetics and Functional Genomics, Center for Functional Genomics of Microbes, University of Greifswald, Greifswald, Germany; 14 Center for Thrombosis and Hemostasis (CTH), Johannes Gutenberg University Medical Center, Mainz, Germany; 15 Irish Centre for Vascular Biology, School of Pharmacy and Biomolecular Sciences, Royal College of Surgeons in Ireland, Dublin, Ireland

## Abstract

Blood coagulation is essential for stopping bleeding but also drives thromboembolic disorders. Factor XII (FXII)–triggered coagulation promotes thrombosis while being dispensable for hemostasis, making it a potential anticoagulant target. However, its physiological role remains unclear. Here, we demonstrate that FXII-driven coagulation enhances innate immunity by trapping pathogens and restricting bacterial infection in mice. *Streptococcus pneumoniae* infection was more severe in FXII-deficient (*F12*^−/−^) mice, with increased pulmonary bacterial burden, systemic spread, and mortality. Similarly, *Staphylococcus aureus* skin infections and systemic dissemination were exacerbated in *F12*^−/−^ mice. Reconstitution with human FXII restored bacterial containment. Plasma kallikrein amplifies FXII activation, and its deficiency aggravated *S. aureus* skin infections, similarly to *F12*^−/−^ mice. FXII deficiency impaired fibrin deposition in abscess walls, leading to leaky capsules and bacterial escape. Bacterial long-chain polyphosphate activated FXII, triggering fibrin formation. Deficiency in FXII substrate factor XI or FXII/factor XI co-deficiency similarly exacerbated *S. aureus* infection. The data reveal a protective role for FXII-driven coagulation in host defense, urging caution in developing therapeutic strategies targeting this pathway.

## Teaser

Factor XII drives thrombosis but is dispensable for hemostasis, making it an anticoagulant drug target. This study shows that factor XII–triggered coagulation traps pathogens and limits bacterial dissemination, highlighting a role of prothrombotic mechanisms in innate immunity and host defense.

## Graphical Abstract

**Figure:**
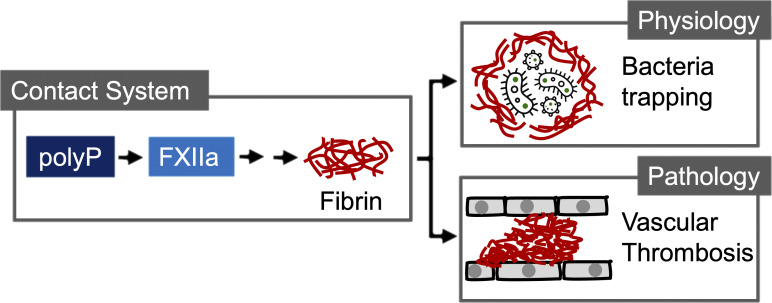

## Introduction

The blood coagulation system consists of a group of tightly regulated plasma proteases, leading to the production of fibrin, which stops bleeding at sites of vascular injury (hemostasis). However, fibrin is also a key component of thrombi that occlude blood vessels and underlie thromboembolic diseases such as ischemic stroke, myocardial infarction, and pulmonary embolism—together representing the leading causes of death in the developed world (Townsend et al., 2022). Fibrin formation can be initiated by two distinct mechanisms, triggered by either exposure of blood to a damaged vessel wall (extrinsic pathway) or by blood-borne factors (intrinsic pathway). The intrinsic pathway of coagulation begins when factor XII (FXII) binds to anionic surfaces, triggering its conversion to the enzymatically active form, FXIIa. In the classical cascade model, FXIIa drives fibrin production via its substrate, factor XI (FXI). Alternatively, FXI can be activated by FXII-independent mechanisms (“feedback activation”).

FXII-driven coagulation was first recognized as essential for surface-activated diagnostic blood coagulation assays (e.g., the activated partial thromboplastin time), which are commonly used as a clinical measure of global plasma coagulation. Despite its critical role in fibrin production in the test tube, for decades FXII was thought to have no function in coagulation in vivo (Long et al., 2016). This premise was based on the fact that individuals deficient in FXII have a normal hemostatic capacity and do not exhibit increased bleeding. Unexpectedly and challenging the dogma of the coagulation balance, “pathological” fibrin formation, and thrombosis were defective in animal models of hereditary FXII deficiency (Renné et al., 2005). FXII-deficient mice were protected in models of thromboembolic diseases, such as ischemic stroke (Kleinschnitz et al., 2006), deep vein thrombosis (DVT), and pulmonary embolism (Grover et al., 2020; Meng et al., 2021).

Based on the critical role of FXII in thrombosis while sparing hemostasis, the protease has emerged as a promising target for safer anticoagulant drug development (Kalinin, 2021). FXII(a)-blocking antibodies (Larsson et al., 2014; Xu et al., 2024), cyclic peptide inhibitors (Wilbs et al., 2020), knockdown of FXII expression by antisense oligonucleotides (ASO) and small interfering RNAs have been developed and shown to provide thromboprotection without bleeding risk in mouse and large animal models of thrombosis (Liu et al., 2020; Revenko et al., 2011; Umei et al., 2023; Yau et al., 2014). However, despite the pathological function of FXII in thrombosis, deficiency in the protease is rare in humans, suggesting that FXII-triggered coagulation may have an important physiological function.

Polyphosphate (polyP) is a linear polymer consisting of two to several thousand orthophosphate units connected by energy-rich phosphoanhydride bonds. In bacteria, polyP serves as a phosphate reservoir and energy storage (Müller et al., 2019). In mammalian blood, extracellular polyP triggers procoagulant responses, including the activation of FXII through contact, acceleration of factor V activation, disruption of fibrinolysis, interference with the anticoagulant function of tissue factor (TF) pathway inhibitors (TFPIs), and a significant amplification of FXI feedback activation (Müller et al., 2009; Morrissey and Smith, 2015; Mailer et al., 2019).

PolyP-activated FXII cleaves plasma prekallikrein (PK) to generate active plasma kallikrein (PKa), which in turn reciprocally activates additional FXII molecules. The amplification loop culminates in a burst of FXIIa activity. Conversely, deficiencies in FXII or PK impair or largely delay contact-induced coagulation (Kearney et al., 2024).

In addition to coagulation, FXIIa activates the bradykinin-producing proinflammatory kallikrein–kinin system (reviewed by Nickel and Renné [2012]). Experimental and clinical data have linked FXIIa and the kallikrein–kinin system to bacterial infections (Herwald et al., 1998; Isenring et al., 2018; Kahn et al., 2002; Sriskandan et al., 2000; Wuillemin et al., 1995).

Gram-positive *Staphylococcus aureus* (*S. aureus*) and *Streptococcus pneumoniae* (*S. pneumoniae*) infections present a major clinical burden. *S. aureus* is one of the most common causes of skin and soft tissue infections and endocarditis (Lowy, 1998), while *S. pneumoniae* (the pneumococcus) is a leading cause of upper and lower respiratory tract infections. Both *S. aureus* and pneumococcal infections are associated with activated coagulation, and upregulated expression of certain coagulation factors (i.e., acute phase response) is a hallmark of bacterial infection. Abscess walls with fibrin deposits are typical of staphylococcal and streptococcal infections and contribute to bacterial trapping and host defense (Cheng et al., 2011; Kobayashi et al., 2015).

Here, we investigated pneumococcal lung and staphylococcal skin infections in mice deficient in FXII-initiated procoagulant pathways. Our findings demonstrate that deficiency in FXII-driven coagulation enhances bacterial infection severity and dissemination. In contrast, fibrin formation mediated by the polyP–FXII-FXI axis supports abscess wall integrity and facilitates bacterial entrapment. These results reveal a physiological role for FXII-driven coagulation in innate immune defense against bacterial pathogens.

## Results

### Increased *S. pneumoniae* dissemination and pneumonia lethality in FXII-deficient mice

**Figure 1.**
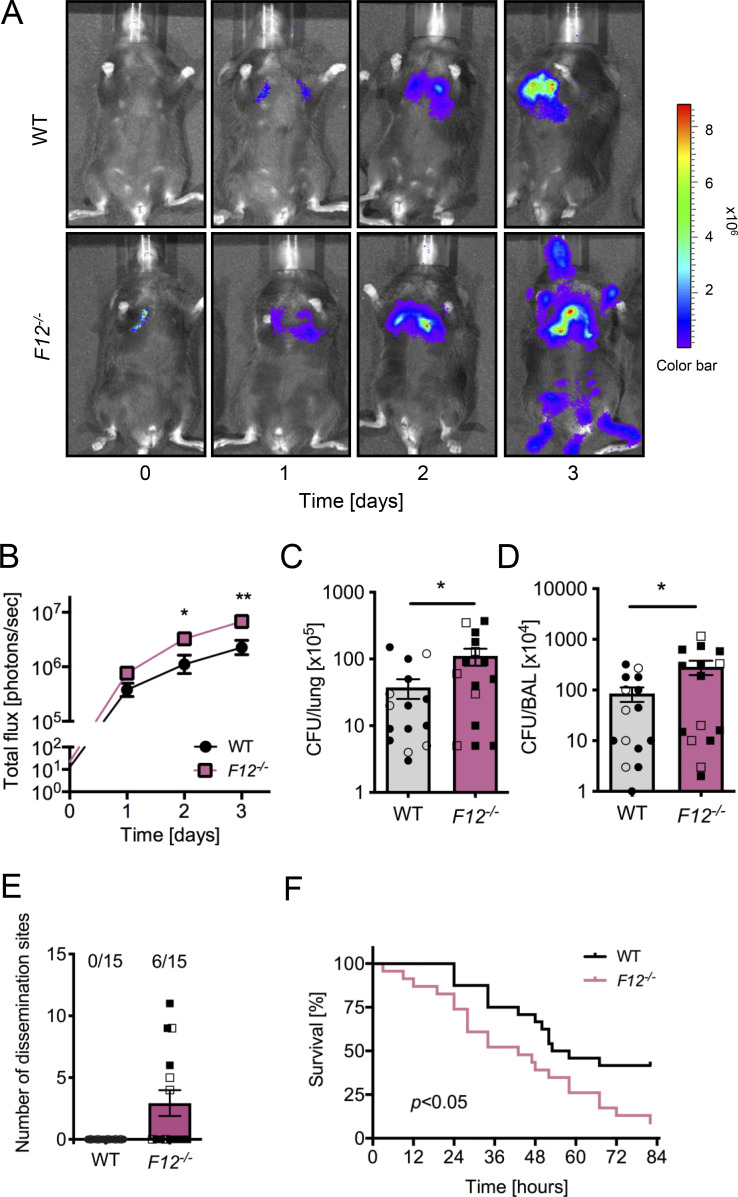
***S. pneumoniae*–induced lethality is increased in *F12*** ^***−/−***^ **mice.** WT and *F12*^*−/−*^ mice were intranasally inoculated with 5.0 × 10^6^ CFUs of *S. pneumoniae* (PN122) Xen10 (*n* = 15 per group comprising 10 male and 5 female mice). **(A)** Representative *S. pneumoniae* in vivo bioluminescence on a pseudocolor scale overlaid on top of a greyscale image of WT and *F12*^*−/−*^ mice. **(B–D)** (B) Bacterial counts as measured by in vivo bioluminescence of *S. pneumoniae* demonstrated as mean total flux (photons per second) ± SEM presented on a logarithmic scale. Bacterial loads observed in (C) lungs and (D) bronchoalveolar lavage (BAL). Filled and empty symbols represent male and female mice, respectively. **(E)** Number of secondary infections detected via *S. pneumoniae* in vivo bioluminescence. **(F)** Survival analysis of WT and *F12*^*−/−*^ mice challenged with *S. pneumoniae*. *P < 0.05 and **P < 0.01 *F12*^*−/−*^ versus WT. P values were determined using one-way ANOVA (B) or Student’s *t* test (C and D).

To investigate the role of FXII in host defense, we used a lethal pneumonia model driven by intranasal inoculation of FXII-deficient (*F12*^*−/−*^) and WT mice with bioluminescent *S. pneumoniae* (strain Xen10, a serotype 3 derivate of strain A66). In vivo bioluminescence correlates closely with bacterial CFUs (Miller et al., 2006). Bacterial growth was accelerated in *F12*^−/−^ mice compared with WT controls, resulting in a higher bacterial burden in challenged lungs (6.8 ± 1.0 × 10^6^ versus 2.3 ± 0.8 × 10^6^ photons/sec on day 3, P < 0.01; Fig. 1, A and B). Consistent with a more severe lung infection, bacterial load was significantly higher both in lung tissues (111 ± 32 × 10^5^ versus 37 ± 12 × 10^5^ CFU; P < 0.05 versus WT; Fig. 1 C) and bronchoalveolar lavage fluid (288 ± 90 × 10^4^ versus 85 ± 27 × 10^4^ CFU; P < 0.05 versus WT; Fig. 1 D) of challenged *F12*^−/−^ mice compared with WT controls. In all WT mice (15 of 15), bioluminescence signals remained restricted to the primary site of infection, the lung. In contrast, pneumococci disseminated in *F12*^−/−^ mice, with bioluminescence signals detected at peripheral sites beyond the lungs in 6 out of 15 challenged mice (Fig. 1 E). Dissemination of pneumococci was associated with significantly higher lethality. At 82 h after infection, 22 of 24 *F12*^−/−^ mice had died, whereas only 14 of 24 WT mice succumbed during this period (P < 0.05 versus WT; Fig. 1 F).

### Increased *S. aureus* skin infection and bacterial dissemination in FXII-deficient mice

**Figure 2.**
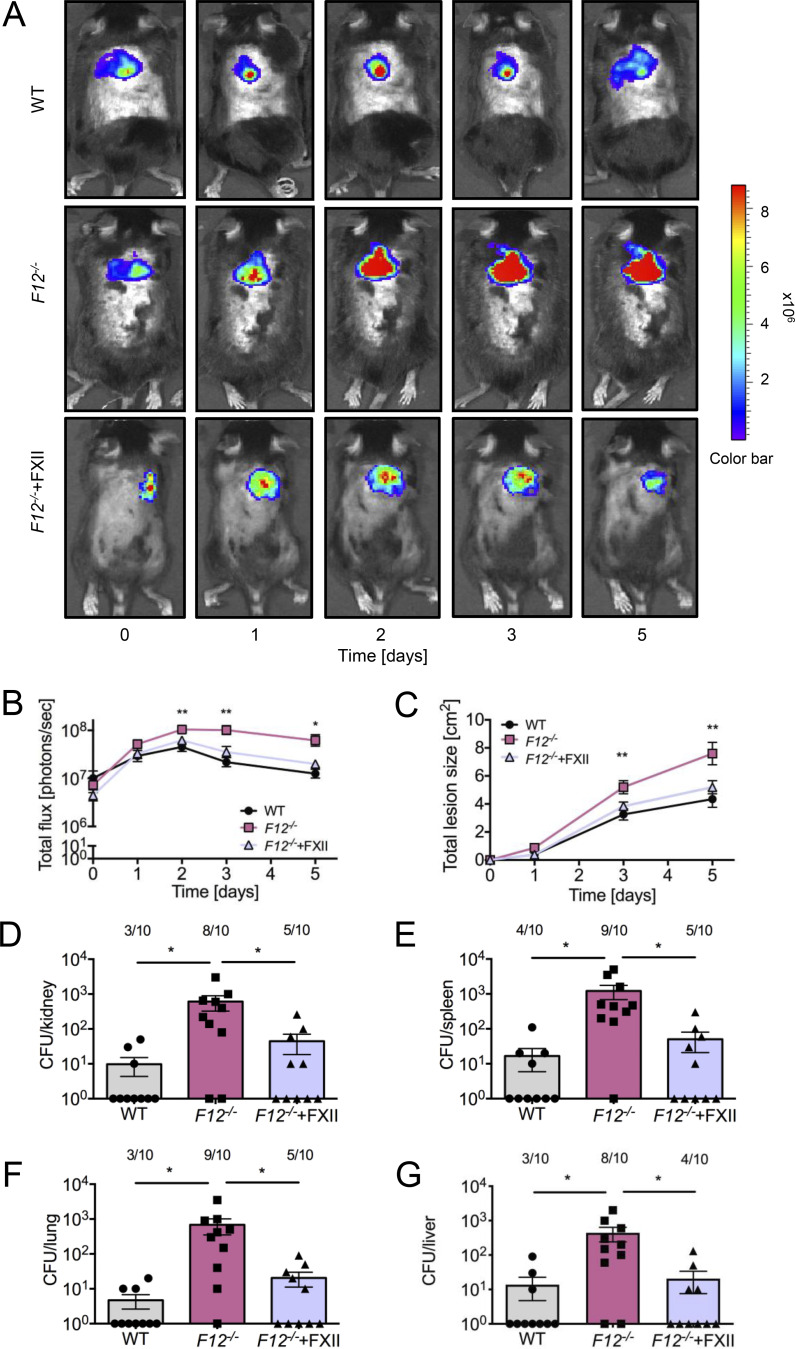
**Accelerated *S. aureus*–induced skin infection in FXII-deficient mice is rescued by human FXII.** WT, *F12*^*−/−*^ mice, and *F12*^*−/−*^ mice reconstituted once with a dose of 2 µg human FXII per gram of body weight (*F12*^*−/−*^+FXII) were injected subcutaneously with 1 × 10^9^ CFU *S. aureus* bioluminescent strain Xen29 (*n* = 10 per group). **(A)** Representative *S. aureus* in vivo bioluminescence on a pseudocolor scale overlaid on top of a greyscale image of WT, *F12*^*−/−*^, and *F12*^*−/−*^+FXII mice. **(B)** Bacterial counts as measured by in vivo bioluminescence of *S. aureus* demonstrated as mean total flux (photons per second) in a logarithmic scale. **(C)** Mean total size of the skin lesion in cm^2^ ± SEM. **(D–G)** Bacterial burdens observed in kidneys (D), spleens (E), lungs (F), and livers (G) of WT, *F12*^*−/−*^, and *F12*^*−/−*^+FXII mice at day 5 after infection. Values are CFUs ± SEM within the entire organ as determined by serial dilutions of tissue homogenates. *P < 0.05 and **P < 0.01 versus WT (B and C) or *F12*^*−/−*^ (D–G). P values were determined using Student’s *t* test (B and C) or one-way ANOVA (D–G).

To demonstrate that FXII deficiency confers an increased risk of bacterial infection severity and pathogen dissemination, we challenged WT and *F12*^−/−^ mice in a skin infection model by subcutaneously inoculating animals with bioluminescent *S. aureus* (Malachowa et al., 2013). In WT animals, infections progressed but peaked by day 2 after challenge, after which bacterial bioluminescence signals decreased. In contrast, bacterial loads reached significantly higher levels in *F12*^−/−^ animals (10.4 ± 0.8 × 10^7^ versus 4.5 ± 1.1 × 10^7^ photons/sec for *F12*^−/−^ versus WT animals at day 2, P < 0.001) and remained nearly constant after day 2, resulting in greater than fivefold higher bacterial counts at day 5 compared with WT mice (1.3 ± 0.3 × 10^7^ versus 6.2 ± 1.8 × 10^7^, P < 0.05; Fig. 2, A and B). Consistent with bioluminescent signal intensities, macroscopic skin wounds developed more rapidly and were significantly larger in *F12*^−/−^ mice compared with WT controls (7.6 ± 0.8 versus 4.3 ± 0.6 cm^2^ at day 5, P < 0.01 versus WT; Fig. 2 C).

To test whether human FXII has a similar importance in entrapping infections as the mouse homolog and to confirm that the increased infection severity observed in *F12*^−/−^ animals was specifically due to absence of FXII and not a secondary effect of the deficiency state, we reconstituted *F12*^−/−^ mice with human FXII (2 µg/g body weight) to normal plasma levels (Kleinschnitz et al., 2006). Reconstitution normalized the prolonged plasma-activated partial thromboplastin time (a measure of FXII-driven coagulation) in *F12*^−/−^ mice (from 69 ± 15 to 30 ± 5 sec), bringing it close to WT levels (28 ± 4 sec, P > 0.05 versus *F12*^−/−^ + FXII). *S. aureus* infections in human FXII-reconstituted *F12*^−/−^ mice developed similarly to those in WT mice (2.0 ± 0.4 × 10^7^ photons/sec at day 5, P > 0.05 versus WT; right panels in Fig. 2, A and B), and the maximum lesion size was comparable with WT controls (5.2 ± 0.5 cm^2^ at day 5, P > 0.05 versus WT; Fig. 2 C).

The high local primary *S. aureus* skin infections facilitate systemic spread of the pathogen (Howden et al., 2023). Therefore, we analyzed mice for bacterial dissemination to peripheral sites. The bacterial load in kidneys (610 ± 285 in *F12*^−/−^ versus 10 ± 5 in WT and 45 ± 26 CFU in *F12*^−/−^ + FXII at day 5, P < 0.05 versus WT), spleens (1,218 ± 535 versus 17 ± 11 and 51 ± 30 CFU, P < 0.05), lungs (682 ± 332 versus 5 ± 2 and 45 ± 26 CFU, P < 0.05), and livers (441 ± 199 versus 14 ± 9 and 21 ± 13 CFU, P < 0.05) was significantly increased in *F12*^−/−^ mice compared with WT mice. In contrast, *S. aureus* disseminated to a similar range in FXII-reconstituted *F12*^*−/−*^ and WT mice, with bacterial counts equally low in all tested organs (FXII versus WT P > 0.05; Fig. 2, D–G).

### Increased *S. aureus* infection and dissemination in PK-deficient mice

**Figure 3.**
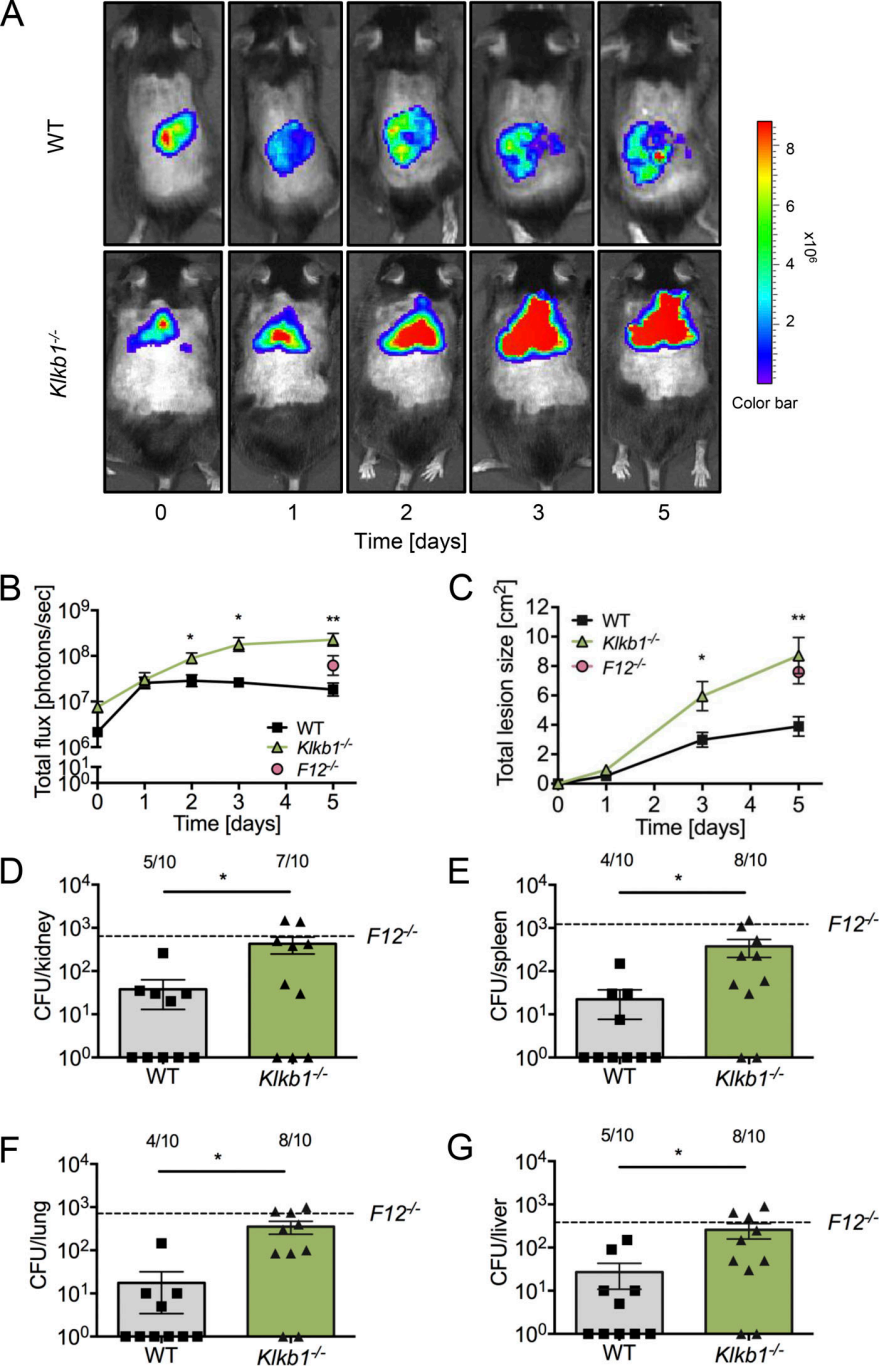
**Increased *S. aureus*–induced cutaneous infection in plasma kallikrein–deficient mice.** WT and *Klkb1*^−/−^ mice were injected subcutaneously with 1 × 10^9^ CFU *S. aureus* bioluminescent strain Xen29 (*n* = 10 per group). **(A)** Representative *S. aureus* in vivo bioluminescence on a pseudocolor scale overlaid on top of a greyscale image of WT and *Klkb*^*−/−*^ mice. **(B)** Bacterial counts as measured by in vivo bioluminescence of *S. aureus* demonstrated as mean total flux (photons per second) ± SEM in a logarithmic scale. **(C)** Mean total size of the skin lesion in cm^2^ ± SEM. **(D–G)** Bacterial burden observed in kidneys (D), spleens (E), lungs (F), and livers (G) of WT and *Klkb1*^*−/−*^ mice at 5 days after infection compared with *F12*^*−/−*^ mice (dashed lines). Values are CFUs ± SEM within the entire organ as determined by serial dilutions of tissue homogenates. *P < 0.05, **P < 0.01 versus WT mice. P values were determined using Student’s *t* test.

The activation of FXII is dependent on PKa, and conversely, PK deficiency impairs FXIIa formation (Kearney et al., 2024). We investigated the effects of PK deficiency (kallikrein B1 gene [*Klkb1*]^−/−^ mice; Sala-Cunill et al., 2015) in our model of *S. aureus*–mediated skin infection. Bacterial bioluminescence signals at the infection site in *Klkb1*^−/−^ mice were significantly elevated compared with WT animals (8.9 ± 2.0 × 10^7^ versus 2.9 ± 1.0 × 10^7^ photons/sec at day 2; P < 0.05 versus WT). While bioluminescence in *Klkb1*^−/−^ mice plateaued on day 3, bacterial burden decreased in WT animals. By day 5 after infection, bacterial load in *Klkb1*^−/−^ mice was over 10-times higher than in WT animals (22.6 ± 9.0 × 10^7^ versus 1.8 ± 0.8 × 10^7^ photons/sec, P < 0.05 versus *Klkb1*^*−/−*^, Fig. 3, A and B). The increased bacterial burden in *Klkb1*^−/−^ mice resulted in more rapid lesion development, with lesion sizes exceeding those in WT mice by more than twofold (8.7 ± 1.2 versus 3.9 ± 0.7 cm^2^ at day 5, P < 0.01 versus WT, Fig. 3 C). There were no statistically significant differences in bioluminescence or lesion size between *Klkb1*^−/−^ and *F12*^*−/−*^ mice. Bacterial spread to peripheral organs was markedly increased in *Klkb1*^−/−^ mice compared with WT animals, as evidenced by significantly higher bacterial burdens in kidneys (429 ± 181 versus 38 ± 25 CFU, P < 0.05 versus WT, Fig. 3 D), spleens (375 ± 167 versus 22 ± 15 CFU, P < 0.05 versus WT, Fig. 3 E), lungs (352 ± 117 versus 18 ± 14 CFU, P < 0.05 versus WT, Fig. 3 F), and livers (203 ± 98 versus 27 ± 16 CFU, P < 0.05 versus WT, Fig. 3 G). The extent of bacterial dissemination in *Klkb1*^−/−^ mice was comparable with that observed in *F12*^*−/−*^ mice (dashed lines indicate mean bacteria load observed in *F12*^*−/−*^ mice, P > 0.05; *Klkb1*^*−/−*^ versus *F12*^*−/−*^). In summary, deficiencies in FXII and PK promote increased *S. aureus* and *S. pneumoniae* growth at the primary infection site and significantly enhance bacterial dissemination to peripheral organs.

### Defective formation of a fibrin abscess capsule facilitates bacterial spreading

**Figure 4.**
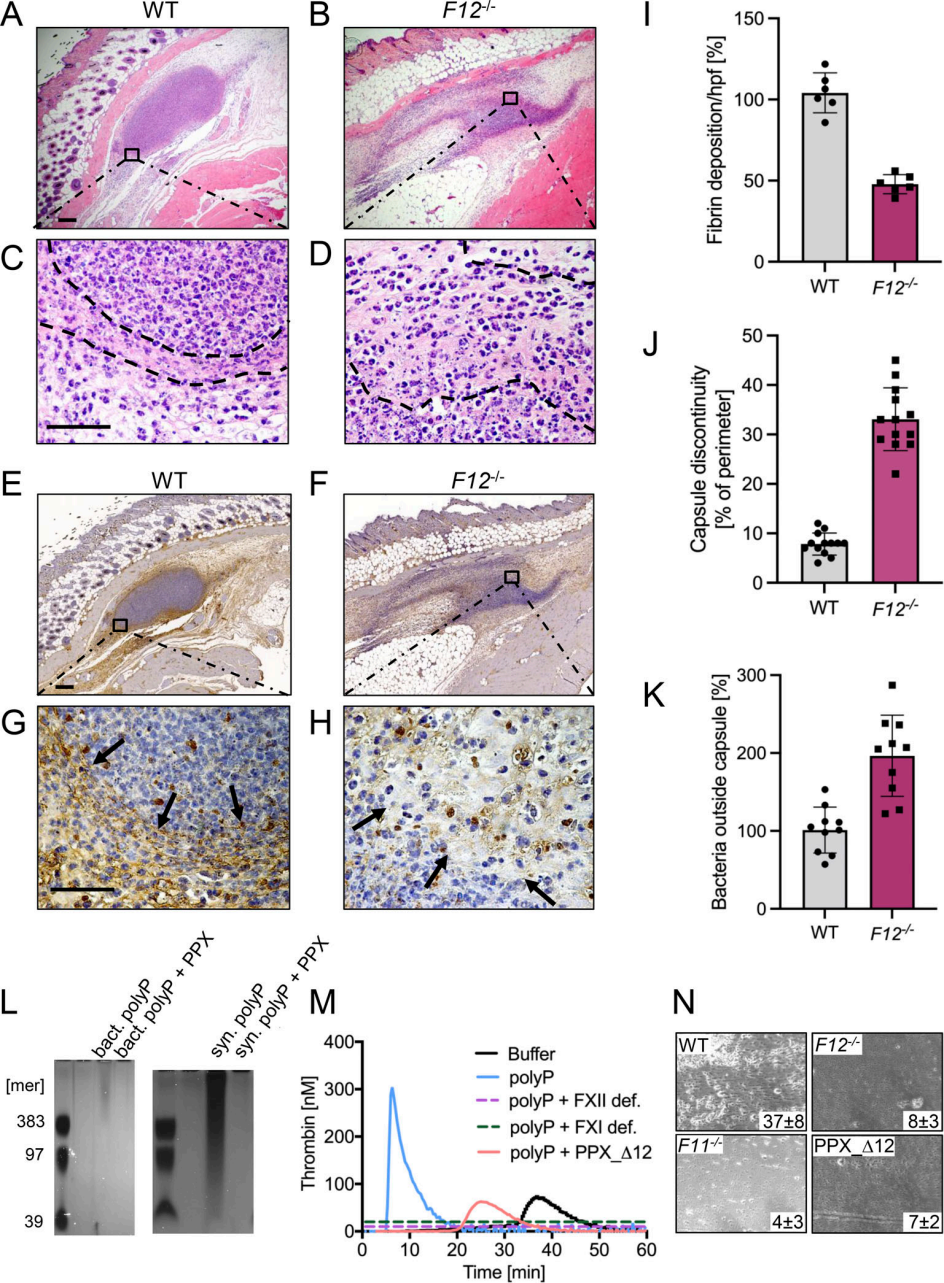
**Defective *S. aureus* polyP-mediated fibrin formation and impaired fibrous abscess capsule integrity in *F12*** ^***−/−***^ **mice. (A–H)** WT, *F12*^*−/−*^ mice were subcutaneously inoculated with 3 × 10^6^ CFU *S. aureu*s, and abscesses were removed 30 h after infection. Representative photographs of skin sections stained with (A–D) H&E or (E–H) fibrin-specific antibody 59D8 are shown. Dashed lines and black arrows denote abscess capsule and fibrin staining around the abscess, respectively. Scale bars represent 50 µm. **(I)** Quantification of fibrin staining given in % relative to WT mice. **(J)** Integrity of abscess capsule measured from immunohistochemistry sections by gaps within the discontinuous fibrin layer of the abscess capsule given relative to the abscess perimeter. **(K)** Number of *S. aureus* detected in the peripheral zone of the abscess given in relative to bacteria counts detected in WT mice, set to 100%. Images were analyzed with ImageJ software. Each symbol represents an individual animal. **(L)** PolyP was extracted from *S. aureus* by anion exchanger chromatography, separated on polyacrylamide/urea gel and visualized by DAPI-negative staining. Synthetic polyP with mean chain lengths of 39, 97, and 383 phosphate monomers served as molecular size standard. Purified polyP was loaded without and after incubation with PPX (10 U/ml for 2 h). A representative gel of *n* = 3 is shown. **(M)** Real-time thrombin generation in normal human plasma stimulated with *S. aureus*–derived polyP in the absence or presence of PPX_Δ12 (1 mg/ml). Plasma deficient in FXII (FXII def.) or FXI (FXI def.) and buffer-stimulated normal plasma was blotted for comparison. Representative thrombin generation curves of a series of *n* = 3. **(N)** Citrated whole mouse blood, readjusted to physiological Ca^2+^ and Mg^2+^ concentrations, was perfused over a surface coated with 3 × 10^6^ CFU *S. aureus* at a venous (100 s^−1^) shear rate. Representative phase-contrast images of thrombi formed during perfusion of WT, *F12*^*−/−*^, *F11*^*−/−*^, or PPX_Δ12 -treated WT blood are shown. Surface covered area by all thrombi in % is given in the lower right corner. A representative experiment of *n* = 3 is shown. Source data are available for this figure: SourceData F4.

Fibrous abscess formation is a hallmark of *S. aureus* skin infections (Kobayashi et al., 2015). A fibrin-rich pseudocapsule functions to trap bacteria and prevents the dissemination of the pathogen (Cheng et al., 2011). FXII has the ability to initiate fibrin formation in pathological thrombosis; conversely, thrombus formation is impaired in *F12*^−/−^ mice (Renné et al., 2005). We hypothesized that FXII-driven prothrombotic mechanisms may also contribute to the formation of an abscess wall. To test this hypothesis, skin abscesses of *S. aureus–*infected *F12*^*−/−*^ and WT mice were histologically compared (Fig. 4, A–H).

In H&E-stained sections of WT mice wounds, a distinct layer of amorphous pinkish material was identified that surrounded bacteria within the abscess core (Fig. 4, A and C). In contrast, this abscess capsule was less defined in *F12*^−/−^ animals (Fig. 4, B and D). To verify the composition and relative quantities of the amorphous material in the abscess wall, we used the antibody 59D8 that specifically binds to fibrin following thrombin-mediated cleavage of fibrinogen and lacks cross-reactivity with the precursor protein (Kleinschnitz et al., 2006). 59D8 immunohistochemistry demonstrated the presence of fibrin deposits along the margins of the *S. aureus* abscess capsules in both WT and *F12*^*−/−*^ mice (Fig. 4, E–H). In comparison to the levels observed in WT animals (which were set to 100 ± 4%), the total fibrin content within the abscess wall was reduced by half (50 ± 3%) in *F12*^−/−^ mice, as determined from the 59D8 immunohistochemical signal intensities (Fig. 4 I).

Consistent with the reduced fibrin deposition observed in the abscess capsule, the overall gap size in the abscess wall, as assessed from histological images was increased by more than threefold in *F12*^*−/−*^ mice compared with WT animals (Fig. 4 J). Defective capsule integrity permitted the spread of *S. aureus* through the abscess wall into surrounding tissues in *F12*^*−/−*^ mice, whereas virtually no bacteria were detectable outside the abscess capsule in WT animals (45.0 ± 7.0 versus 1.0 ± 0.5 per high-power field in *F12*^*−/−*^ versus WT; (Fig. 4 K).

We further investigated the mechanism underlying the initiation of fibrin formation in *S. aureus*–associated abscess capsules. Bacteria contain polyP, which functions as their energy storage pool. We hypothesized that bacterial polyP induces FXII contact activation and triggers FXII/PK-mediated coagulation in plasma. Negative DAPI staining revealed that *S. aureus* contained long-chain polyP with chain length of 400 to >1,000 phosphate monomers. The DAPI-negative signal was susceptible to incubations with recombinant exopolyphosphatase (PPX), an enzyme that specifically cleaves polyP with a chain length greater than >35 phosphate units (Rangarajan et al., 2006), confirming that loaded material purified from bacteria was polyP. Synthetic long-chain polyP was treated identically and loaded as control (Fig. 4 L). *S. aureus*–derived polyP triggered coagulation in normal plasma (Fig. 4 M). In contrast, *S. aureus*–derived polyP was inactive for triggering thrombin production in plasma that is deficient in FXII or the FXIIa substrate FXI. Similarly, thrombin generation in normal plasma was largely reduced when bacterial polyP was preincubated with the recombinant polyP-neutralization probe PPX_Δ12 (Labberton et al., 2016). We analyzed whether the polyP-FXII axis contributes to *S. aureus*–mediated coagulation under more physiological settings in the presence of blood flow. Citrate anticoagulated whole blood was recalcified and then perfused over immobilized *S. aureus* (Fig. 4 N). When WT mouse blood was perfused over *S. aureus* bacteria, thrombi formed within 7 min from the time of perfusion (37 ± 8% surface covered area). In contrast, deficiency in FXII or FXI or addition of the polyP inhibitor PPX_Δ12 largely reduced *S. aureus*–driven thrombus formation to 8 ± 3%, 4 ± 3%, and 7 ± 2%, respectively. Taken together, the data show that *S. aureus* polyP has the capacity to initiate coagulation via the polyP-FXII axis in plasma.

### The FXII–FXI pathway contributes to abscess wall integrity in *S. aureus* infections

**Figure 5.**
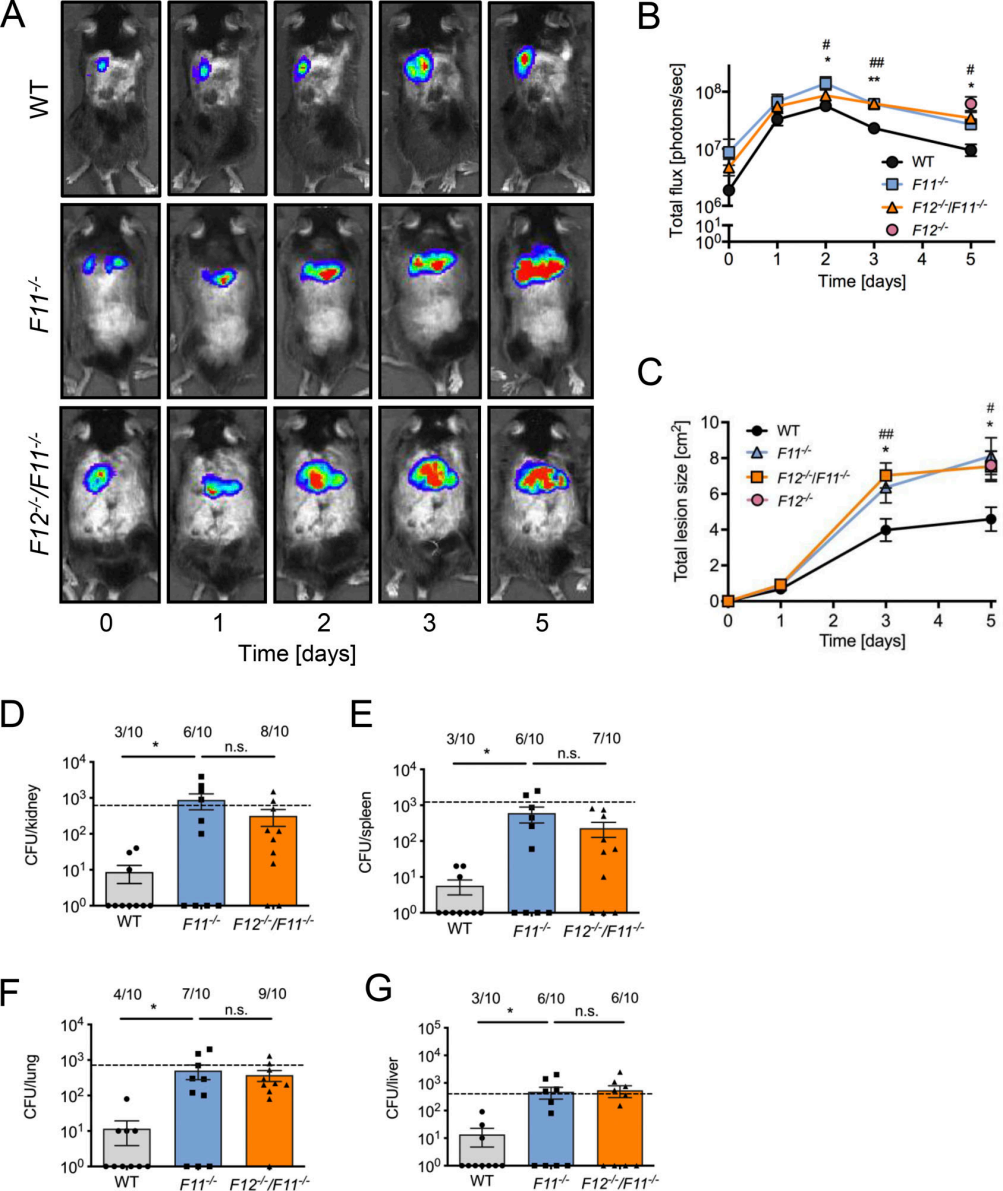
**FXII-FXI–driven coagulation traps *S. aureus* in skin infection. (A)** WT, *F11*^*−/−*^, or *F12*^*−/−*^*/F11*^*−/−*^ mice were injected subcutaneously with 1 × 10^9^ CFU *S. aureus* bioluminescent strain Xen29 (*n* = 10 per group). Representative *S. aureus* in vivo bioluminescence on a pseudocolor scale overlaid on top of a greyscale image of WT, *F11*^*−/−*^, or *F12*^*−/−*^*/F11*^*−/−*^ mice are shown. **(B)** Bacterial counts as measured by in vivo bioluminescence of *S. aureus* demonstrated as mean total flux (photons per second) ± SEM in a logarithmic scale. **(C)** Mean total size of the skin lesion in cm^2^ ± SEM. *P < 0.05, **P < 0.01 *F11*^*−/−*^ versus WT mice and ^#^P < 0.05, ^##^P < 0.01 *F12*^*−/−*^*/F11*^*−/−*^ versus WT mice in A. **(D–G)** Bacterial burden observed in kidneys (D), spleens (E), lungs (F), and livers (G) of WT, *F11*^*−/−*^, and *F12*^*−/−*^*/F11*^*−/−*^ mice compared with *F12*^*−/−*^ mice (dashed lines) at day 5 after infection. Values are CFUs ± SEM within the entire organ as determined by serial dilutions of tissue homogenates. *P < 0.05 versus WT mice. P values were determined using Student’s *t* test.

PolyP initiates procoagulant activity in vitro by two alternative mechanisms: (1) by amplifying activated FXI-mediated TFPI inactivation (Puy et al., 2016) and (2) by inducing FXII contact activation leading to FXI activation (Müller et al., 2009). To identify the mechanism by which FXII mitigates *S. aureus* skin infection severity and pathogen dissemination, we used FXI-deficient (*F11*^*−/−*^) and *F12*^*−/−*^/*F11*^*−/−*^ double gene-deficient mice (Müller et al., 2009). If fibrin production for *S. aureus* encapsulation is mediated by FXII activation and the subsequent activation of FXI by FXIIa, then *F11*^*−/−*^ and *F12*^*−/−*^/*F11*^*−/−*^ animals should be susceptible to *S. aureus* infections to the same extent as *F12*^*−/−*^ mice. Indeed, skin infection sites were significantly increased in *F11*^*−/−*^ and *F12*^*−/−*^/*F11*^*−/−*^ mice compared with WT animals starting from day 2 and only slightly decreased until day 5 after challenge (2.7 ± 0.5 × 10^7^ and 3.5 ± 1.0 × 10^7^ versus 1.0 ± 0.3 × 10^7^ photons/sec, P < 0.05 versus WT; Fig. 5, A and B). Bacterial bioluminescence signals were similar in *F11*^*−/−*^ and *F12*^*−/−*^/*F11*^*−/−*^ mice and not different from infected *F12*^*−/−*^ animals (P > 0.05 for all comparisons). Skin lesions developed faster and with similar kinetics in infected *F11*^*−/−*^ and *F12*^*−/−*^/*F11*^*−/−*^ compared with WT mice (8.1 ± 1.0 and 7.5 ± 0.8 cm^2^ versus 4.6 ± 0.7 cm^2^, P < 0.05 versus WT). Skin ulcers in *F11*^*−/−*^ and *F12*^*−/−*^/*F11*^*−/−*^ animals were not significantly different in size from those observed in *F12*^*−/−*^ mice (P > 0.05 each; Fig. 5 C). Increased bacterial growth at the injection site was associated with increased bacterial burden, dissemination rates, and severity of infection. Bacterial loads in *F11*^*−/−*^ and *F12*^*−/−*^/*F11*^*−/−*^ mice were significantly higher than those in WT animals in kidneys (879 ± 412, 318 ± 156 versus 9 ± 5 CFU, P < 0.05 versus WT, Fig. 5 D), spleens (603 ± 284, 293 ± 103 versus 6 ± 3 CFU, P < 0.05 versus WT, Fig. 5 E), lungs (501 ± 222, 375 ± 126 versus 12 ± 8 CFU, P < 0.05 versus WT, Fig. 5 F), and livers (478 ± 219, 541 ± 248 versus 14 ± 9 CFU, P < 0.05 versus WT, Fig. 5 G). In all tissues analyzed, bacterial load was similar in *F11*^*−/−*^ and *F12*^*−/−*^/*F11*^*−/−*^ mice and comparable with that in *F12*^*−/−*^ mice (indicated by dashed lines in Fig. 5, D–G).

## Discussion

The innate immune system represents a phylogenetically ancient strategy that provides the first line of defense against invading pathogens. In invertebrates with an open circulatory system, such as arthropods and mollusks, a hemolymph coagulation system captures pathogens, preventing them from entering the host. This ancient cross talk between innate immunity and coagulation is thought to have persisted through evolution (Loof et al., 2011; van der Poll and Herwald, 2014). Experimental and preclinical studies have identified a pivotal role of the FXII–FXI-driven coagulation pathway in pathologic thrombosis but not hemostasis (Chan and Weitz, 2023; Kluge et al., 2022; Müller et al., 2011). The present study shows that coagulation mechanisms underlying pathologic thrombosis may have beneficial functions in host defense by contributing to fibrin abscess capsules formation, thereby limiting bacterial dissemination. Accordingly, deficiency in the FXII–FXI axis impairs contact-driven coagulation and increases the spreading of Gram-positive pneumococci and *S. aureus* (Figs. 1,2, and 5).

Consistent with our data from mouse models, *S. aureus* induces thrombin generation in human plasma via activation of FXII (Loof et al., 2015; Mattsson et al., 2001). Multiple clinical studies have shown that FXII is activated and consumed in plasma during Gram-negative sepsis (Duan et al., 2023; Levi et al., 2003; Wuillemin et al., 1995). Furthermore, FXII activation is associated with poor prognosis in infected patients with overt disseminated intravascular coagulation (DIC) (Park et al., 2016). Although it is difficult to conclude from case reports, the medical history of Mr. John Hageman, the index patient for FXII deficiency, supports a role for FXII in host defense. Mr. Hageman had a lifelong history of recurrent sinusitis that is commonly caused by *S. aureus* infections (Ratnoff, 1980).

Consequences of FXII–FXI-driven coagulation in bacterial infections depend on the localization of coagulation activation. At primary sites of infection in the skin (Figs. 2 and 5) and lung (Fig. 1), fibrin production mediated by the FXII–FXI axis provides protection by fibrous abscess capsule wall formation. In contrast, once the pathogen has entered the circulation, FXII–FXI-driven coagulation becomes detrimental. Procoagulant reactions triggered by disseminated bacteria in the blood stream contribute to DIC. Indeed, intravenous infusion of heat-inactivated *S. aureus* (Silasi et al., 2021) or live *Escherichia coli* (Jansen et al., 1996; Pixley et al., 1993) induces severe thrombo-inflammatory responses in an FXII-dependent manner in nonhuman primates. Infection severity was consistently increased in our *F11*^*−/−*^ and *F12*^*−/−*^/*F11*^*−/−*^ mice following intravenous administration of live *S. aureus* (data not shown). Conversely, targeting FXII in the circulation with inhibitory antibodies improves survival (Pixley et al., 1993). Similarly, infusion of FXI-neutralizing antibodies improves survival following polymicrobial peritoneal sepsis induced by cecal ligation and puncture (Tucker et al., 2012). Taken together, FXII–FXI-driven coagulation contributes to the entrapment of these two Gram-positive cocci at the site of infection. The procoagulant mediator polyP has been detected in a wide range of bacterial species, including *S. aureus*, the SL-1 *Streptococcus* strain, and *S. pneumoniae*, with levels reaching up to 30% of bacterial dry weight in *Acinetobacter johnsonii* (Tanzer and Krichevsky, 1970; Deinema et al., 1985; Kulaev and Vagabov, 1983; Bowlin and Gray, 2021). The polymer’s chain length and content are subject to dynamic regulation by activities of polyP kinases PPK1 and PPK2, and PPX, the phosphate regulon, and responses to environmental stresses (Lamarche et al., 2008). Adding to this complexity, platelets and other blood-circulating cells release polyP (Müller et al., 2009; Moreno-Sanchez et al., 2012; Verhoef et al., 2017), while polymers from disintegrating host lung and skin cells may also contribute to FXII contact activation (Kumble and Kornberg, 1995). Furthermore, FXI can be activated independently of FXII via TF-triggered thrombin, which is further amplified by polyP (Choi et al., 2011), suggesting a complex role for polyP in FXII–FXI and TF–thrombin–FXI pathways involved in bacterial containment. However, if bacteria escape and enter the circulation, the coagulation pathway can go awry, culminating in DIC.

To escape coagulation-based entrapment in a host, bacteria express plasminogen activators (Pla's), which have been shown to support pathogen dissemination (McDevitt et al., 1997; Sun et al., 2004). In particular, the plague agent *Yersinia pestis* has the ability to disrupt clots by activating the host fibrinolytic system (Sun et al., 2004). *Yersinia* species express a surface Pla that facilitates bacterial dissemination. Targeting Pla, however, results in effective control of the infection, an increased presence of inflammatory cells at the infection sites, and significantly improved survival rates. Other pathogens, including pneumococci and *S. aureus* express enzymes that activate the host fibrinolytic system (Wang et al., 1998), disrupt the integrity of the abscess wall, and enable *S. aureus* dissemination in skin infection models (Bergmann and Hammerschmidt, 2007; Peetermans et al., 2014).

Consistent with natural bacterial polyP (Fig. 4), synthetic polyP of the size produced by bacterial triggers FXII-dependent coagulation (Zilberman-Rudenko et al., 2018). In addition to driving coagulation by activating host FXII, *S. aureus* has the ability to induce fibrin production via its endogenous coagulases staphylocoagulase and von Willebrand factor-binding protein. These bacterial proteins exert procoagulant activity via direct activation of prothrombin that in turn leads to the formation of a staphylothrombin complex. Staphylothrombin converts fibrinogen into fibrin (Friedrich et al., 2003), driving fibrous abscess capsule formation (McAdow et al., 2012). Formation of fibrin by staphylothrombin provides an explanation for residual fibrin deposits in FXII-deficient mice (Fig. 4, F and H). The relative importance of FXII–FXI and staphylocoagulase pathways appears to depend on the site of infection, bacterial strain, and phase of infection. In a rabbit skin and soft tissue infection model, fibrin formation of the abscess capsule was mediated by coagulases independently of the FXII–FXI axis (Malachowa et al., 2016). Similarly, a preceding study of *S. pneumoniae* lung infection in mice found that bacteria-triggered coagulation occurred via FXI independently of FXII (Stroo et al., 2017). The discrepancy between the findings of Stroo et al. (2017) and those of the present study is likely due to methodological variations, including differences in infection severity, experimental design, and bacterial strain selection. Specifically, Stroo and colleagues employed a 10-fold lower bacterial inoculation dose (5 × 10^5^ CFU/mouse), which has been shown to induce less TF expression compared with the doses used in the present study (Levi et al., 2003). The relative contribution of FXIIa-versus TF-mediated thrombin-driven FXI feedback activation depends on TF activity (von dem Borne and Meijers, 1995). Real-time thrombin generation assays have shown that FXI feedback activation plays a pivotal role in coagulation at low TF concentrations, whereas at higher TF levels, coagulation activation becomes predominantly independent of the FXI feedback mechanism (Kravtsov et al., 2009). This renders FXI feedback activation particularly relevant to the experimental design of Stroo and colleagues. In a low-grade *E. coli* LPS-induced endotoxemia model in humans, FXI was consistently activated by thrombin, independently of FXIIa (Minnema et al., 1998). Furthermore, Stroo et al. (2017) used a *S. pneumoniae* serotype 2 (D39), and the present study employed a serotype 3 strain (Xen10). Because capsule serotypes influence numerous aspects of pneumococcal pathogenesis, e.g., resistance to phagocytosis, survival during infection, colonization, invasiveness, dissemination, and overall disease progression (Geno et al., 2015; Yother, 2011), direct comparisons between different serotypes are challenging. Indeed, in the present study, the majority of *F12*^−/−^ mice succumbed to infection within four days (Fig. 1), whereas animals challenged with lower doses of a different pneumococcus capsule serotype survived beyond 10 days (Stroo et al., 2017).

The mouse models (Fig. 5) and ex vivo plasma (Fig. 4) data indicate that fibrin produced via the polyP–FXII–FXI pathway drive fibrous abscess capsule formation; in contrast, the addition of FXII (physiological levels; 400 nM) did not affect the growth of *S. aureus* or *S. pneumoniae* in culture. Furthermore, neutrophil extracellular traps components, host defense cells, antimicrobial peptides, and FXIIa-produced bradykinin contribute to bacterial entrapping (Bochenska et al., 2015; Oehmcke et al., 2009). To dissect roles of fibrin depositions versus other pathways transgenic mice with nonpolymerizable fibrin (Hur et al., 2024) or analysis of cohorts of individuals with severe FXII deficiency (e.g., <1%) or patients on FXII(a)/FXI(a) inhibitors may provide valuable insights in future studies.

Fibrin helps the host to cope with infection (Mullarky et al., 2005; Prasad et al., 2015; Sun et al., 2004, 2009). Consistently, mice with a factor V Leiden mutation (a prothrombotic risk factor) are protected from bacterial sepsis. Conversely, defective coagulation in a hemophilia A mouse model increases sepsis-induced mortality (Kerschen et al., 2015). In synergy with plasma factors, neutrophils induce coagulation by exposing neutrophil extracellular traps and inactivating the anticoagulant tissue factor pathway inhibitor (TFPI) (Massberg et al., 2010). TFPI-mediated amplification of TF-driven extrinsic coagulation activity interferes with extravasation of *E. coli* strain XA90 (Massberg et al., 2010), whereas this prothrombotic mechanism appears not to be sufficient to combat *S. aureus* and pneumococci in the absence of FXII, despite the substantial recruitment of leukocyte to the abscess wall (Fig. 4).

PKa liberates bradykinin from high molecular weight kininogen and amplifies FXII activation (Kearney et al., 2024). ASO-mediated knockdown of kininogen gene expression attenuates inflammatory reactions, bacterial dissemination, and growth in multiple organs in a *Streptococcus pyogenes* mouse sepsis model. Depletion of kininogen in mice was associated with a partial PK deficiency, impeded streptococcal spreading, and reduced inflammatory reactions (Köhler et al., 2020). Consistently, the small molecule D-Pro-Phe-Arg chloromethyl ketone (PCK that inhibits PKa>>FXIIa); (Tans et al., 1987) interferes with *Salmonella typhimurium*–triggered contact system activation in lethal experimental sepsis (Persson et al., 2000). In contrast, in our *S. aureus* skin infection model, a complete deficiency in PK augmented local bacterial growth and dissemination. Together, the data may reflect fundamental differences upon residual PKa activities in the knockdown and pharmacologic models that are absent in gene knockout mice or bacterial strain-specific differences in their procoagulant and fibrinolytic activities. It is recommended that subsequent studies utilize a range of pharmacological agents that target the polyP/FXII/FXI/PK pathway (Chan and Weitz, 2023; Mailer et al., 2022; Vappala et al., 2024) to analyze the effects of complete pathway inhibition, as observed in the gene-knockout mouse lines employed in this study, in comparison to transient interference with residual activity on infection severity and pathogen dissemination.

Based on potent thromboprotection while sparing hemostasis observed in experimental thrombosis models in mice and large animals, the polyP–FXII–FXI axis has emerged as a novel target for “safe anticoagulants” (Fredenburgh et al., 2017). Available X-ray structure and epidemiological data have facilitated development of FXI-blocking agents, including neutralizing antibodies, ASO/small interfering RNAs, and small molecule inhibitors (reviewed by Bentounes et al. [2023]; Fredenburgh and Weitz [2023]; Gailani and Gruber [2024]). Initially, ASO-mediated inhibition of *F11* expression in humans has been shown to provide protection against DVT (Büller et al., 2015). Currently, many novel “safe” anticoagulants that selectively interfere with FXI/FXIa (milvexian, asundexian, abelacimab, IONS fesomersen, REGN-9933) (Verstraete et al., 2024) are in phase 2 and 3 clinical trials for the prophylaxis of atrial fibrillation, DVT, or secondary stroke. These treatments are close to entering clinical practice with far-reaching medical and economic implications for millions of patients worldwide (Chan and Weitz, 2023; Cohen and Ageno, 2022; Fredenburgh and Weitz, 2023; Kluge et al., 2022; Piccini et al., 2025). In addition, long-term pharmacologic neutralization of FXIIa (garadacimab) (Cohn and Renné, 2024) is emerging as prophylactic therapy for hereditary angioedema, a bradykinin-mediated swelling disease (Craig et al., 2023). If the role of FXII–FXI-driven coagulation in bacterial infections translates to humans, pharmacologic interference with FXII(a) and FXI(a) might increase risk of bacterial infection severity and pathogen dissemination leading to sepsis in treated patients, which should be monitored cautiously.

## Materials and methods

### Bacteria strains and growth conditions

Bioluminescent *S. aureus* strain Xen29 and *S. pneumoniae* strain Xen10 are genetically engineered isolates of their parental strains *S. aureus* ATCC 12600 and *S. pneumoniae* A66.1 (NCTC 7978) that express a modified *Photorhabdus luminescens* lux operon (Francis et al., 2001; Kadurugamuwa et al., 2003). Both strains are capable of producing both the luciferase enzyme and its substrate, thereby constitutively emitting a bioluminescent signal when the organisms are metabolically active. The strains were obtained commercially from Xenogen (Caliper Life Science) and have a kanamycin resistance plasmid selection marker. *S. aureus* was cultured in lysogeny broth (LB) medium and *S. pneumoniae* in Todd–Hewitt medium supplemented with 0.5% yeast extract. Cultures were grown to OD_600_ ∼0.6 (*S. aureus*) or OD_600_ ∼0.4 (*S. pneumoniae*) and washed three times with either sterile saline or sterile PBS/1% FCS, respectively.

### Mice

All animal care and experimental procedures complied with the 3R Principles of Laboratory and Animal Care established by the National Society for Medical Research, were conducted in compliance with local authorities, and were approved by Institutional Review Boards, including the Bezirksregierung of Unterfranken (54–2531.01-17/06; Würzburg; *S. pneumoniae* lung infections), Regierung of Oberbayern (55.2-1–54–2531-8-07; Munich; *S. pneumoniae* lung infections), and Stockholm’s Norra Djurförsöksetiska Nämnd (N427/10, N246/13, N129/11, N57/14; *S. aureus* skin infection). *F12*^−/−^ (Pauer et al., 2004), *F11*^−/−^ (Gailani et al., 1997), *Klkb1*^−/−^ (Sala-Cunill et al., 2015) mice were backcrossed for >10 generations to the C57Bl/6J background, as previously described. *F12*^−/−^ and *F11*^*−/−*^ mice were bred to generate *F12*^+/−^/*F11*^+/−^ mice. These animals were crossed to generate *F12*^−/−^/*F11*^−/−^ double-deficient mice with the expected Mendelian ratio. Western blotting confirmed absence of FXII and FXI in *F12*^−/−^/*F11*^−/−^ mice. Progenies were genotyped by using PCR. All experimental animals used in these studies were sex- and aged-matched (both male and female). Age- and sex-matched WT control mice were purchased from Charles River (Wiga).

### Blood collection

Human plasma was obtained from healthy informed volunteers at the Karolinska University Hospital, approved by the Stockholm ethical committee (Regionala Etikprövningnämden). Peripheral venous blood was collected into 3.2% trisodium citrate (9:1 blood-to-citrate ratio). The first 10 ml of sample was discarded. Platelet-poor plasma was prepared by two consecutive centrifugation steps at 3,000 × *g* for 10 min each. Plasma from individuals with inherited FXII and FXI deficiency was obtained from George King Bio-Medical, Inc. Factor levels were below the western blot detection limit. For blood collection, mice were anesthetized by intraperitoneal injection of 2,2,2-tribromoethanol and 2-methyl-2-butanol prior to being subjected to retro-orbital blood sampling. Murine blood was collected into 3.8% trisodium citrate.

### Thrombus formation under flow

Thrombus formation under flow was analyzed as previously described (Larsson et al., 2014) with some minor modifications. Briefly, coverslips were coated with *S. aureus* (1 × 10^6^ CFU/cm^2^) and blocked with HEPES buffer (136 mM NaCl, 2.7 mM KCl, 0.42 mM NaH_2_PO_4_, 5 mM HEPES, 2 mM MgCl_2_, and 1% BSA, pH 7.4) for 30 min. Coverslips were placed onto a transparent, 50-μm deep parallel-plate flow chamber (Maastricht Instruments BV), which was pre-rinsed with BSA-containing buffer. Chambers were co-infused with citrate-anticoagulated blood and isotonic CaCl_2_/MgCl_2_ solution (10:1 ratio) using pulse-free pumps, resulting in free Ca^2+^ and Mg^2+^ concentrations of ≈2 mM each. In some cases, blood samples were spiked with PPX_Δ12 (500 μg ml^−1^). After 7 min of flow (shear rates of 100 s^−1^ for venous flow conditions), flow chambers were rinsed with HEPES buffer (pH 7.4) containing 2 mM CaCl_2_. Phase-contrast images were recorded with an ORCA-Flash 2.8 CMOS camera (Hamamatsu) and Nikon Eclipse Ti microscope equipped with a ×20 objective. The surface area covered by thrombi was calculated in each image using ImageJ 4.0 software.

### Preparation of *S. aureus* for skin inoculation and cutaneous infection

A single colony of the *S. aureus* strain Xen29 was used to inoculate LB medium overnight at 37°C. Overnight cultures were diluted 1:50 into fresh LB medium, grown to the mid-logarithmic phase (OD_600_ ∼0.6), washed three times, and were resuspended in sterile pharmacy-grade saline (0.9%) at the indicated concentration. CFUs per milliliter were verified by colony counts of serial dilutions plated on LB agar. In the cutaneous infection model, the skin of mice was shaved on the back. Mice were anesthetized using 5% isoflurane, and the back skin was inoculated subcutaneously with *S. aureus* (1.0 × 10^9^ CFU) in 100 μl sterile saline (0.9%). Measurement of total lesion size was measured manually using a millimeter ruler. Mice were euthanized at day 5 after infection. For histological analysis, mice were subcutaneously inoculated with 3.0 × 10^6^ CFU, and abscesses were collected 30 h after infection.

### *S. pneumoniae* mouse pneumonia model

Intranasal infections with bioluminescent *S. pneumoniae* and monitoring of pneumococcal growth and dissemination in real-time in mice were performed as described previously (Saleh et al., 2014).

### Quantification of bacteria by in vivo bioluminescence

Mice were anesthetized using isoflurane. In vivo bioluminescence imaging was performed using the Xenogen IVIS imaging system (Caliper Life Sciences). Total photon emission of each mouse was quantified with the Living Image software package (Caliper Life Sciences) as previously described (Saleh et al., 2013). Bioluminescence intensities are presented on a pseudocolor scale superimposed on a grayscale photograph. Data were quantified as total flux (photons/sec) within the region of interest.

### Bacterial burden and microscopic analysis of tissues

At selected time points after infection, mice were anesthetized by intraperitoneal injection of 2,2,2-tribromoethanol and 2-methyl-2-butanol, and organs were harvested for analyses. The organs were excised and homogenized in ice-cold PBS. Aliquots were serially diluted, plated on LB agar plates and incubated for determining CFU. In addition, abscesses of mice inoculated with 3 × 10^6^ CFU *S. aureus* were fixed in 4% PFA for 48 h at room temperature, embedded in paraffin and thin-sectioned. Sections were either stained with H&E or analyzed histologically for fibrin presence. For fibrin detection, sections were incubated in antigen retrieval buffer (pH 6.2; Biocare Medical) and heated in a 2100 Antigen Retriever (Aptum Biologics Ltd.) for 20 min. Endogenous peroxidases were blocked by incubation in 3% hydrogen peroxide for 20 min. Nonspecific-binding sites in murine tissue sections were blocked using blocking serum provided in the mouse on mouse staining kit (Vector Laboratories). Sections were incubated with 4.7 µg/ml 59D8 antibody (Kleinschnitz et al., 2006; Renné et al., 2005; von Brühl et al., 2012) in the presence of 1% BSA and 0.01% Tween for 1 h at room temperature. Fibrin was detected using the mouse on mouse staining kit (Vector Laboratories) using 3, 3′-diaminobenzidine as a substrate. Slides were briefly counterstained in Mayer’s hematoxylin (Biocare Medical) and photographed on a NIKON Eclipse E600 microscope. Measurement of fibrin deposition/abscess was made by analyzing digital photographs using ImageJ software (Version 2.0). Leukocytes and bacteria per visual field were counted at 40× magnification from 6 high-power fields (hpf) of H&E-stained sections.

### PolyP isolation and detection

PolyP was extracted from *S. aureus* as previously described (Nickel et al., 2016) with the following modification: Bacteria were lysed with 100 µg/ml lysostaphin in 20 mM Tris (pH 7.4) in the presence of a phosphatase inhibitor cocktail (Sigma-Aldrich) for 15 min at 37°C before adding sulfuric acid (0.3 M) and sodium chloride (3.5 M), and DNA was digested with 100 µg/ml DNaseI (Sigma-Aldrich) in the presence of 3.5 mM MgCl_2_ for 1 h at 37°C before proteinase K (750 µg/ml) digestion for an additional hour. The final polyP preparation was eluted in 20 mM Tris (pH 7.4). PolyP was separated by PAGE using 10% polyacrylamide tris-borat-EDTA (TBE)-urea (7 M) gels and stained using DAPI-negative staining as previously described (Smith and Morrissey, 2007).

### Real-time thrombin generation analysis

Thrombin formation in real-time was analyzed with the calibrated automated thrombography method using a Fluoroscan Ascent fluorometer (Thermo Fisher Scientific) equipped with a dispenser (Thrombinoscope BV) as previously described (Nickel et al., 2015). In brief, synthetic (P30; Israel Chemicals Ltd.) or bacterial polyP was incubated with buffer (Tris 20 mM, pH 7.4), PPX, or PPX_Δ12 for 30 min at 37°C (Labberton et al., 2016). Samples were then added to 80 μl platelet-poor plasma. Thrombin generation was initiated by adding 2.5 mM fluorogenic substrate (ZGGR-AMC; Thrombinoscope BV), and all experiments were run in triplicate. Thrombin formation was quantified using the Thrombinoscope software package (Version 3.0.0.29).

### Statistical methods

Normal distribution was determined by a quantile–quantile plot, and data were analyzed by unpaired two-tailed Student’s *t* test or, in the case of multiple comparisons, one-way ANOVA followed by post hoc analysis using Tukey’s multiple comparisons test. Prism 8.0 (GraphPad) was used for analysis, and values of P < 0.05 were considered statistically significant. Data are expressed as mean values ± SD, unless indicated otherwise.

## Supplementary Material

SourceData F4 is the source file for Fig. 4.

## Data Availability

Data are available in the article itself and its supplementary materials. Additional information will be provided upon request from the corresponding author via email: thomas@renne.net.
