# Supplementary figures and images for: Factor XII–driven coagulation traps bacterial infections
Source: J Exp Med. 2025 Apr 22;222(7):e20250049. doi: 10.1084/jem.20250049 (PMC12013512; doi:10.1084/jem.20250049)

Left panel: Figure 4L

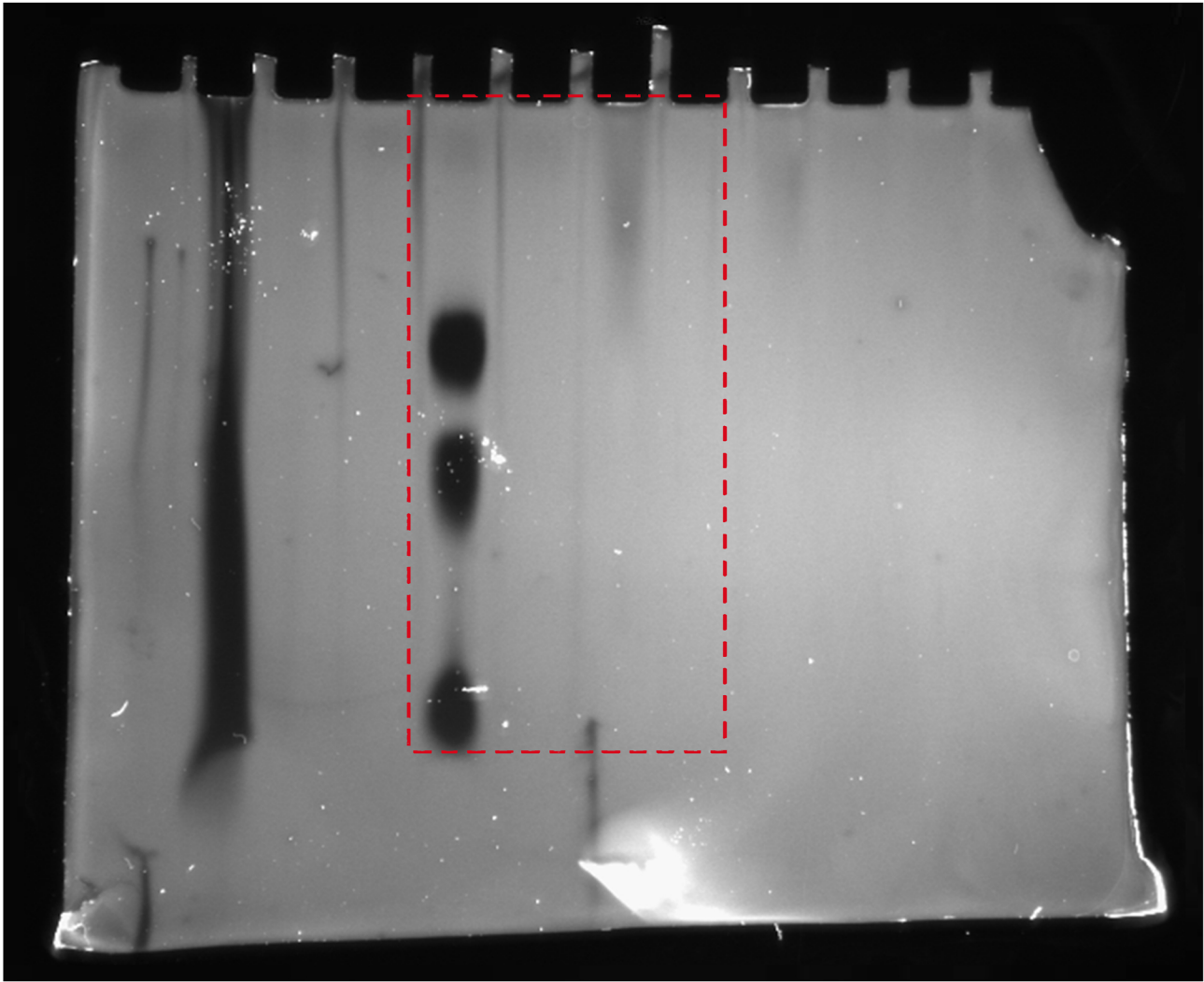

Right panel: Figure 4L

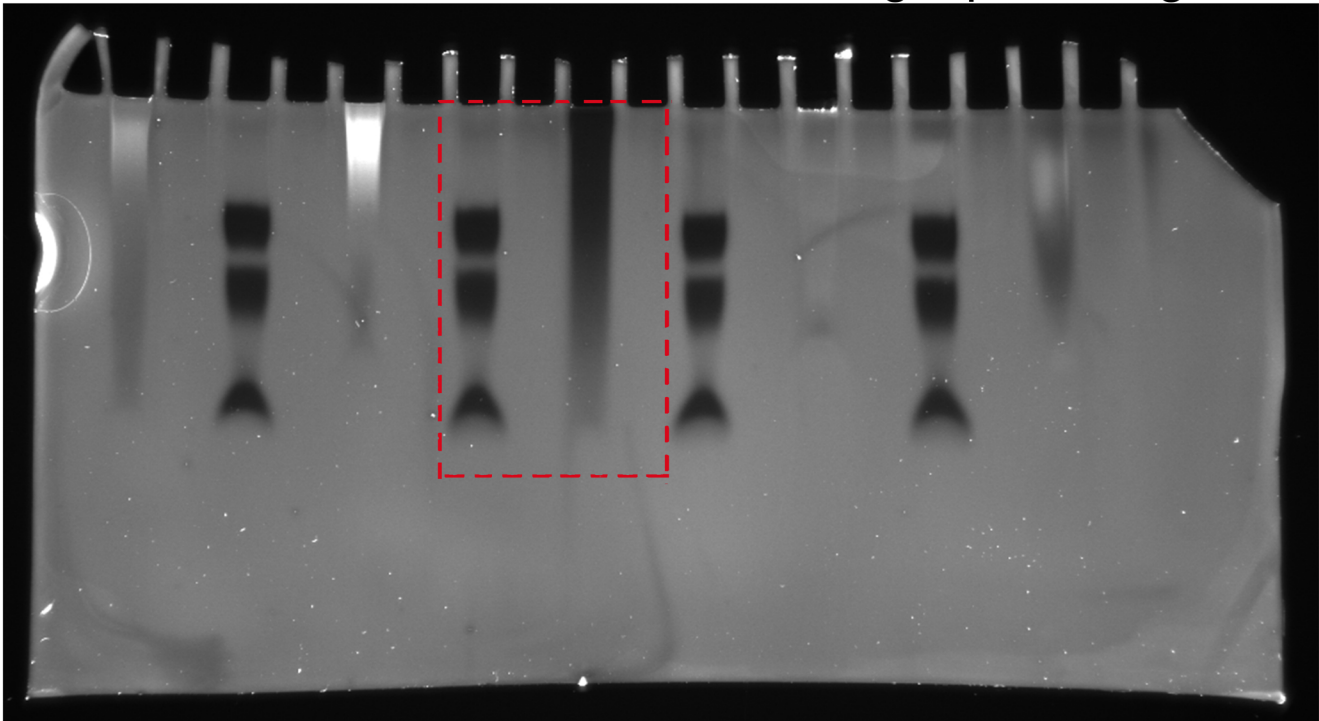

Supplement: SourceData F4 — is the source file for Fig. 4. [file jem_20250049_sourcedataf4.pdf]
